# Linking suckling biomechanics to the development of the palate

**DOI:** 10.1038/srep20419

**Published:** 2016-02-04

**Authors:** Jingtao Li, Chelsey A. Johnson, Andrew A. Smith, Daniel J. Hunter, Gurpreet Singh, John B. Brunski, Jill A. Helms

**Affiliations:** 1 Department of Oral and Maxillofacial Surgery, West China Hospital of Stomatology, Sichuan University, Chengdu, China 610041; 2 Division of Plastic and Reconstructive Surgery, Department of Surgery, Stanford School of Medicine, Stanford CA 94305; 3 Department of Plastic Surgery, University of Pittsburgh Medical Center, Pittsburgh, PA 15261.

## Abstract

Skulls are amongst the most informative documents of evolutionary history but a complex geometry, coupled with composite material properties and complicated biomechanics, have made it particularly challenging to identify mechanical principles guiding the skull’s morphogenesis. Despite this challenge, multiple lines of evidence, for example the relationship between masticatory function and the evolution of jaw shape, nonetheless suggest that mechanobiology plays a major role in skull morphogenesis. To begin to tackle this persistent challenge, cellular, molecular and tissue-level analyses of the developing mouse palate were coupled with finite element modeling to demonstrate that patterns of strain created by mammalian-specific oral behaviors produce complementary patterns of chondrogenic gene expression in an initially homogeneous population of cranial neural crest cells. Neural crest cells change from an osteogenic to a chondrogenic fate, leading to the materialization of cartilaginous growth plate-like structures in the palatal midline. These growth plates contribute to lateral expansion of the head but are transient structures; when the strain patterns associated with suckling dissipate at weaning, the growth plates disappear and the palate ossifies. Thus, mechanical cues such as strain appear to co-regulate cell fate specification and ultimately, help drive large-scale morphogenetic changes in head shape.

Because eating is essential to surviving, structures involved in feeding are often under extreme selective pressure, leading to the evolution of a wide range of vertebrate craniofacial morphologies. For example, the acquisition of a hinged jaw joint some 420 million years ago^1^ allowed gnathostomes to expand into previously unavailable trophic niches, including those associated with prey capture and mastication^2^,^3^,^4^. The palate has experienced similar adaptive changes^5^. In basal clades including protostomes the oral and nasal cavities are contiguous, there is no palate, and feeding occurs primarily by filtration of organisms or organic particles from water^6^. Fish^7^ and birds^8^ develop palatal shelves but these incompletely separate the oral and nasal cavities and both have evolved independent mechanisms (e.g., pharyngeal teeth^9^ and gizzards^10^, respectively) for grinding food into digestible particles.

On the other hand, mammals and some reptiles including alligators and crocodiles have palatal shelves that completely separate the oral and nasal cavities^11^. This arrangement has many advantages; for example, a contiguous hard palate plays a mechanical role in stabilizing the rostrum during mastication and thus allows for an enormous radiation of feeding mechanisms^12^,^13^,^14^. Possessing a palate that separates the nose from the mouth also allows *Crocodilia* to maintain their typical lurking position, i.e., breathing with submerged jaws. The mammalian palate also provides a surface against which the tongue can manipulate food during mastication and be buttressed during sucking^15^. Before they begin to chew, mammalian feed by suckling^16^ which requires an intact palate^17^,^18^. The anterior portion of the palate supports the tongue as it cups around the teat to express milk, while the posterior portion closes off the nasal cavity and allows the generation of negative pressure to ensure that milk enters the esophagus^19^. Palatal clefting results continuous nasal and oral cavities and this anatomical defect precludes the generation of negative pressure by suckling^20^.

We propose that suckling is more than just an important means of feeding. We hypothesize that the strains generated by this mammalian-specific oral behavior correspond to gene transcription and cell differentiation programs that influence palatal development and impact craniofacial morphogenesis. Indirect support for such a mechanobiological-based theory comes from a number of in vitro^21^,^22^ and in vivo studies^23^,^24^ that collectively show how physical stimuli (e.g., compression, tension, stress, strain) can regulate gene transcription and, correspondingly, tissue growth and development. Using finite element (FE) modeling, we mapped the patterns of strain caused by suckling and tongue movements onto the anatomy of the developing prenatal palate. An unexpected result emerged, where hydrostatic and distortional strains favored the formation of cartilage in a site of intramembranous ossification. Thus, mechanical cues during a distinctively mammalian-specific feeding behavior appeared to drive an unexpected change in cell fate specification.

## Results

**Figure 1:**
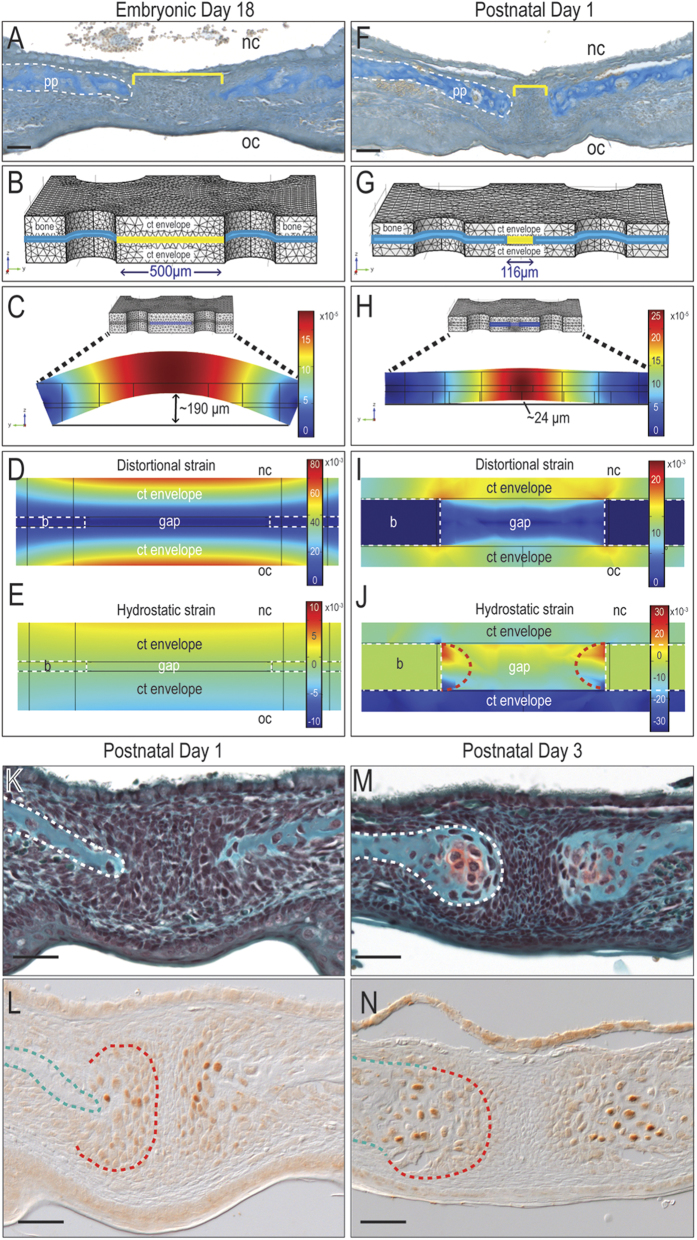
Postnatal mechanical environment predicts the emergence of cartilage in the midpalatal suture. Aniline blue staining of representative tissue sections from (**A**) E18 and (**F**) P1 mouse palate. Meshed three-dimensional model of (**B**) E18 and (**G**) P1 mouse palate. The extents of deflection (**C,H**), distortional strain (**D,I**) and hydrostatic strain (**E,J**) were mapped. (**K,M**) Safranin O/Fast green staining and (**L,N**) Sox9 immunostaining on representative sections through P1 and P3 mouse palate. Abbreviations: pp = palatal process; b = bone; ct = connective tissue; nc = nasal cavity; oc = oral cavity. Scale bar = 50 μm. Note: all images are oriented with the nasal surface of the palatal bone facing upwards, and the oral surface facing downwards.

Mammalian suckling begins in utero^25^; therefore, our mechanobiological analyses began at embryonic day 16.5 (E16.5), soon after the bilateral palatal processes that comprise the palate have fused. An FE model was constructed to understand how physical forces associated with suckling affected the prenatal palatal tissues. Histology (Fig. 1A) and micro-computed tomography (μCT) anatomical data guided the geometry of the embryonic palate structure in the FE model (Fig. 1B and Table 1). Mechanical properties were then assigned to the materials in the structure: for example, the fibrous tissue between the developing palatal processes was treated as a homogenous, linearly elastic material (Table 2) based on molecular and cellular analyses demonstrating a relatively uniform, undifferentiated population of proliferating cranial neural crest cells (Supplementary Fig. 1). Based on published data, loading and displacement conditions from suckling pressures and tongue forces^26^,^27^ were prescribed for various surfaces of the palatal structure.

Distortional and hydrostatic strains were computed using COMSOL software in order to capture how suckling and tongue movements affected shape changes and volume changes, respectively, in tissues in the developing palate. The overall deflection of the palate was also computed. Suckling created a net downward pressure and tongue movements a net upward force on the prenatal palate; coupled with a relatively low resistance to deformation, the combined mechanical loads caused a major (190 μm) deflection of the ~500 μm thick prenatal palate (Fig. 1C). In the fibrous tissue-filled gap between the bony palatal processes, the magnitude and distribution of distortional (Fig. 1D) and hydrostatic strains (Fig. 1E) were fairly homogeneous (Fig. 1D,E).

The forces associated with suckling increased dramatically during the postnatal period, when neonates initiate nursing^27^. The palatal processes also thickened; therefore, a second FE model was used to map the predicted strains with this scenario (Fig. 1F). A quantitative analysis of postnatal palatal architecture was performed and these histomorphometric data guided the assignment of new tissue geometries. For example, relative to their dimensions at E18.5, the postnatal day 1 palatal bones (blue) were thicker; the connective tissue envelope was thinner; and the fibrous tissue-filled gap between the bony palatal processes (yellow) was narrower (Fig. 1G). When these new geometries were subjected to the significantly larger pressures and tongue forces associated with nursing, minimal deflection of the palate was observed (Fig. 1H). This relative rigidity (Fig. 1I) led to the creation of hydrostatic strains that were particularly high (e.g., 3%) at the edges of the palatal bones (dotted semi-circular red lines, Fig. 1J).

Distortional and hydrostatic strains of this magnitude (Fig. 1I,J) were consistent with the specification of chondrogenic cell fate^28^,^29^,^30^. However, the prediction of cartilage in the developing palate was surprising. According to a well-accepted literature, the palatal processes form exclusively through the direct differentiation of mesenchymal cells into osteoblasts (reviewed in^31^). Our own histological analyses from late embryonic stages to post-natal day 1 also showed that cells in the palatal processes directly differentiate into osteoblasts (Fig. 1K and Supplementary Fig. 2).

**Figure 2:**
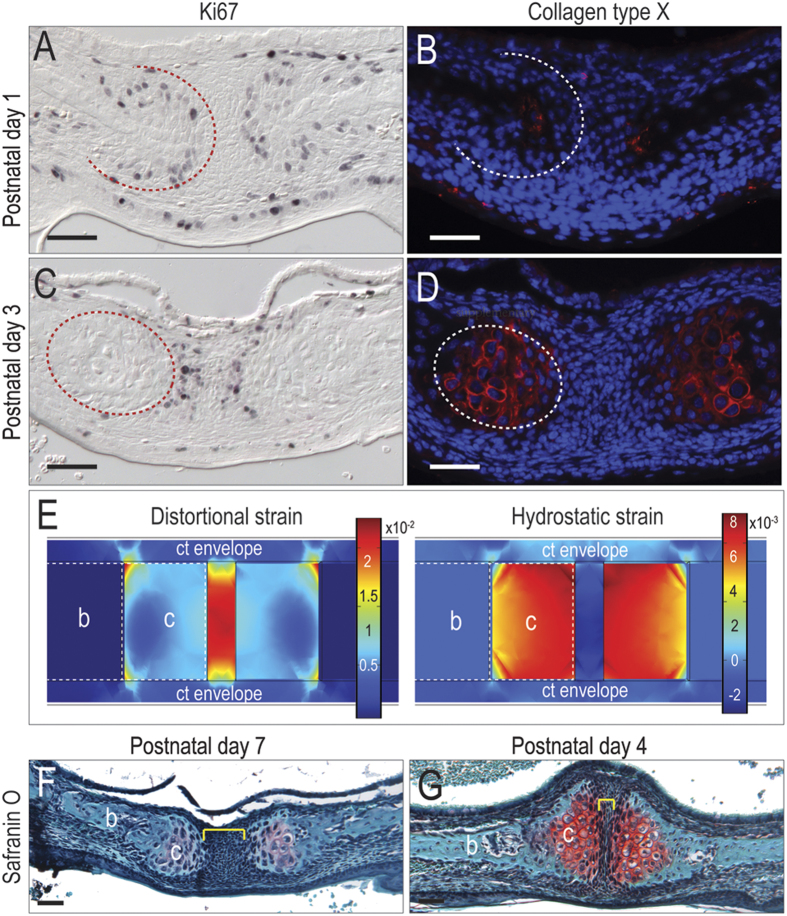
The cartilage caps at the midpalatal suture become hypertrophic soon after their emergence. (**A**) At postnatal day 1, Ki67 activity is detected on the outer rim of a grouping of cells (outlined in red) capping the palatal processes with the midline devoid of staining. (**B**) The expression of collagen type X is only detected at the leading edge of the palatal shelves. (**C**) By postnatal day 3, Ki67 staining is positive only at the leading edge of each palatal process and absent in the midline (outlined in red), whereas (**D**) collagen type X expression is detected between the proliferating cells and palatal process edge (outlined in white). (**E**) Distortional and hydrostatic strain distribution were mapped while applying an expansion force in the cartilage cap. (**F**) Safranin O/Fast green staining at P4 with the palatal process separated by an unorganized region of mesenchymal cells (yellow bracket). (**G**) By P7 the midpalatal suture complex is organized into a proteoglycan-rich cartilage matrix (red in Safranin O/Fast green staining) region at the end of the bony palatal process and the midline area is now narrowed. Abbreviations: b = bone; c = cartilage; ct = connective tissue. Scale bar = 50  μm.

Based on the FE prediction of cartilage formation, however, we interrogated the palatal tissue using immunostaining for the pre-chondrogenic protein Sox9^32^. These analyses revealed that discrete regions of cells, localized to the edges of the palatal bone, were Sox9-positive (Fig. 1L). We focused on the perinatal period and carefully mapped the onset of Sox9 expression; Sox9 protein was absent at e18.5, became detectable on P1 and by P3 a proteoglycan-rich, cartilaginous matrix was obvious (Fig. 1M,N). In this 72 hour window of time, the chondroprogenitor cells transitioned from highly proliferative chondroblasts (Fig. 2A,B) to fully differentiated, hypertrophic chondrocytes (Fig. 2C,D). Thus, for reasons that were not immediately clear, the cranial neural crest cells that differentiated to form the palatal bones had suddenly shifted from an osteogenic to a chondrogenic program.

To understand how this dramatic shift in cell fate status impacted subsequent palatal morphogenesis we incorporated these cartilage “caps” into an iterative FE model. When a group or condensation of cells is growing faster than surrounding cells, the volume of the condensation is constrained, which in turn leads to an increase in pressure in the condensation^28^. In our model, chondrogenic cells were increasing in size (hypertrophying) faster than surrounding cells, as demonstrated by collagen type X-positive immunostaining for hypertrophic chondrocytes. To simulate this state of hypertrophy in the FE model the chondrogenic cells were assigned a uniform pressure^29^. Postnatal suckling pressure and tongue force were then applied. Collectively these conditions produced high hydrostatic strain in the cartilage caps themselves (Fig. 2E). Hydrostatic strains of this magnitude produced a mechanical environment that favors chondrocyte terminal differentiation^28^ on postnatal days 4 and 7 verified the cartilage fate (Fig. 2F,G).

**Figure 3:**
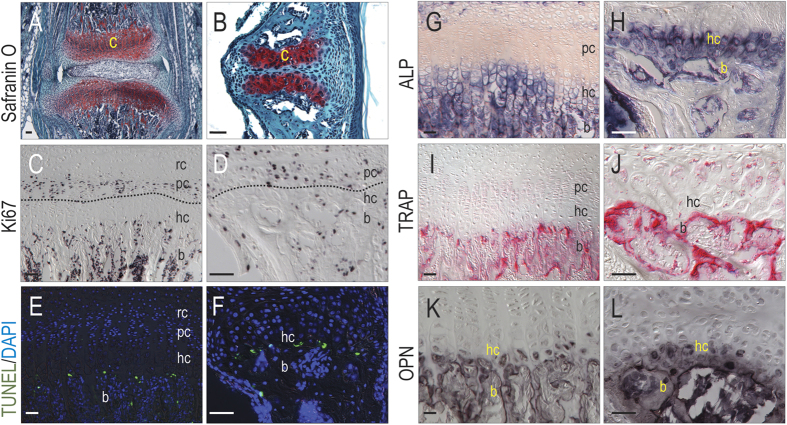
The midpalatal suture complex is analgous to the vertebral growth plate. (**A**) Representative sections at P21 with Safranin O/Fast green staining showing two regions of organized proteoglycan-rich cartilage matrix separated by the intervertebral disc of the vertebral growth plate and (**B**) the fibrous tissue in the midpalatal suture complex. (**C**) Zones of Ki67^+ve^ chondrocytes are identified both in the vertebral growth plate (**D**) and the midpalatal suture. (**E**) TUNEL^+ve^ cells are detected at the chondro-osseous junction in the growth plate of both the vertebra and (**F**) palate. (**G**) Positive ALP staining is detected in the region of the hypertrophic and ossifying chondrocytes of the growth plate in both the vertebra and (**H**) palate. (**I**) TRAP^+ve^ cells are most apparent throughout the bone of both the vertebra and (**J**) palate. (**K**) Osteopontin^+ve^ staining was detected in the hc region of the growth plate as well as the bone in both the vertebra and (**L**) palate. Abbreviations: ALP = alkaline phosphatase; b = bone; c = cartilage; hc = hypertrophic chondrocytes; OPN = Osteopontin; pc = proliferating chondrocytes; rc = resting chondrocytes; TRAP = tartrate-resistant acid phosphatase; TUNEL = terminal deoxynucleotidyl transferase-mediated dUTP nick end labeling; Scale bars = 50 μm.

The cartilage caps resembled growth plates (Fig. 3A,B), because there was an obvious proliferative zone (Fig. 3C,D), an apoptotic zone (Fig. 3E,F), and ALP staining in the mineralizing matrix (Fig. 3G,H). TRAP activity (Fig. 3I,J) and osteoblast differentiation (Fig. 3K,L) were consistent with growth plate histology such as is observed in the vertebrae and long bones of mammals^33^. Thus, physical forces generated by a combination of suckling and tongue movements created a strain pattern consistent with the formation of cartilage instead of bone. Furthermore, the chondrocytes organized themselves into growth plate-like structures. These midline growth plates contribute to lateral expansion of the skull^34^.

**Figure 4:**
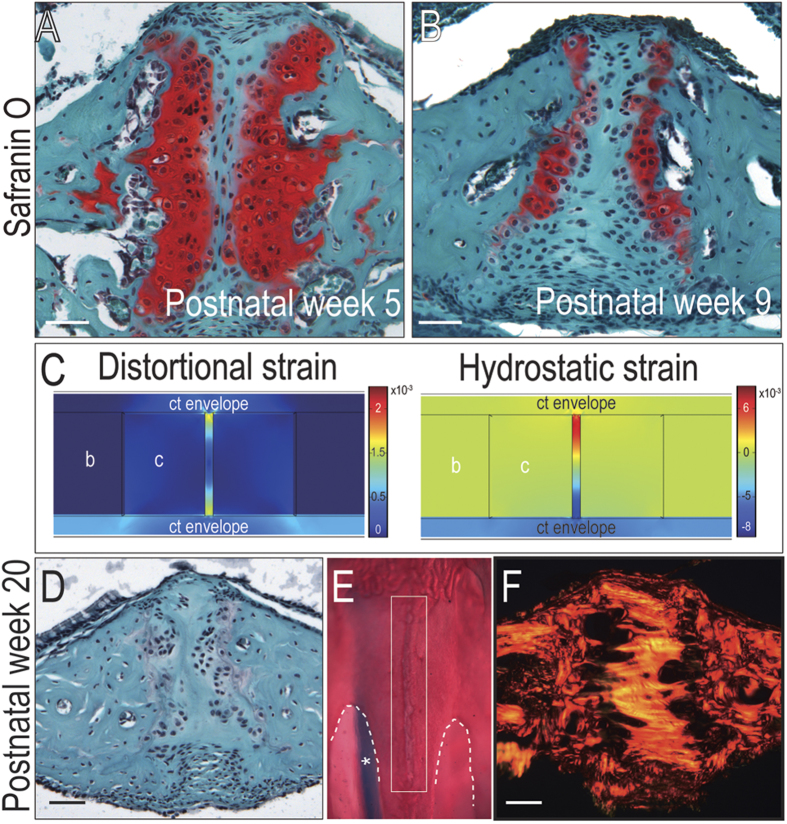
Transient nature of midpalatal suture complex. Safranin O/Fast green staining of representative midpalatal suture sections from 5 week old and (**B**) 9 week old mice. (**C**) As the stiffness of the cartilage cap increases, the distortional and hydrostatic strains are mapped in a corresponding finite element model. (**D**) Safranin O/Fast green staining, (**E**) whole-mount Alizarin red/Alcian blue staining (midpalatal suture outlined in white; asterisk indicates nasal septum), and (**F**) Picrosirius red staining of palate tissue from 20 weeks old mice, indicating loss of cartilage growth plates. Abbreviations: b = bone, c = cartilage, ct = connective tissue. Scale bar = 50 μm.

If pressures generated by suckling are responsible for the emergence and maintenance of these midpalatal growth plates then reduced suckling should result in their disappearance. Mouse pups weaned around 3–4 weeks of age^35^ and we modeled this change in oral behavior, along with other salient anatomical alterations. For example, the gap between the growth plates narrowed and the hypertrophic cartilage became progressively mineralized (Fig. 4A,B). To mimic this observed increasing stiffness, the cartilage was assigned progressively larger elastic moduli (Supplementary Fig. 3). Computed strains in the gap region increased dramatically (Fig. 4C), which was consistent with ossification of the region^36^. By week 20 we found the cartilaginous growth plates had disappeared (Fig. 4D,E) and been replaced by a dense collagen bridge (Fig. 4F). Disappearance of the growth plates thus coincided with the normal cessation of lateral skull growth, which by this age has achieved adult murine dimensions^37^.

## Discussion

The question of how complex tissues are generated from initially homogeneous populations of cells is as old as the field of development biology. While most studies focus on biological stimuli responsible for pattern generation (reviewed in^38^), physical forces can also modify the expression of genes and therefore affect tissue patterning and morphogenesis^39^. Here we provide data to support a mechanobiological model whereby mammalian-specific behaviors create patterns of hydrostatic and distortional strain that correlate with the appearance and disappearance of mid-palatal growth plates that appear to be specific to mammalian palatogenesis.

Abundant data exist on the genetic control of palatogenesis^31^ and embryologists have scrutinized the process of palatal development in great detail. Despite careful searching, however, we were unable to find references to transient growth plate-like structures in the mammalian palatal midline. In the clinical literature a reference was made to the presence of cartilage in a 14-week-old human palate^40^; a more recent experiment study on palatal expansion in 6-week-old mice also showed the presence of cartilage caps at the ends of the bony palatal processes^41^. Neither study identified the cartilage as growth plate-like structures, recognized its transient nature, nor assigned it a role in governing lateral expansion of the face. The importance of these temporary structures is most clearly revealed in a surgical procedure to correct a palatal defect^34^; even if the growth plates themselves are not exposed, scarring in the overlying soft tissues can impede cell proliferation within the growth plates and result in midfacial growth arrest.

If our hypothesis is correct, that suckling creates unique strain patterns that drive the formation of midpalatal growth plates, then mammalian offspring that consume milk by methods other than suckling should lack such transient midpalatal structures. In that regard, monotremes (e.g., platypus and echidna) were of particular interest. In these mammals, lactation is achieved through mammary patches^42^,^43^ and since suckling is thought to require a nipple or teat, it is often assumed that platypus offspring consume milk by licking^44^. If monotreme young lick instead suck- yet still have midpalatal growth plates- then our hypothesis would be incorrect. We examined the palates of monotremes, which are comprised of two bones that fuse in the midline^45^ and also sought to verify how monotreme young actually feed. Archival video data clearly demonstrated echidna and platypus young exhibit suckling behaviors^46^ that is so vigorous as to allow the neonate to consume nearly 10% of its body weight in milk in a single feeding^47^. Therefore, analyses of this Class of mammals neither supported nor refuted our model.

We considered other Classes of animals: If the patterns of hydrostatic and distortional strain created by suckling and tongue movement are unique to mammals then analyses of non-suckling animals with complete bony palates (e.g., crocodiles and alligators) would be quite informative. The theory presented here would predict that *Crocodilia* palates form solely through intramembranous ossification. Multiple hatchling stages would have to be examined, however, since these growth plates are transient structures. There is, however, one caveat: *Crocodilia* may also exhibit other prenatal oral behaviors that influence palatal development.

We wondered if neonates with palatal clefts developed cartilage growth plates. In humans at least, cleft palatal defects do not preclude in utero suckling^48^. Murine fetuses with cleft palates, however, do not survive past birth when the emergence of the growth plates is first observed (Fig. 1). Perhaps in cases of incomplete palatal cleft, this question can be answered by further studies.

The temporal emergence of the cartilage caps precisely corresponds in time to the onset of nursing, when suckling forces increase by an order of magnitude over their prenatal state. We show that the strain patterns generated by these physical forces correlate, temporally and spatially, with discrete patterns of chondrogenic cell fate specification. Chondrocytes organize themselves into bilateral growth plate-like structures that contribute to lateral expansion of the head skeleton. The growth plates are temporary, appearing around the time of weaning. When a neonate transitions from suckling to the mastication of foods, the growth plates disappear and the palate ossifies. Thus, from an initially flexible tissue the palate is converted into a rigid platform, suitable to support diverse masticatory functions. This work emphasizes the importance of physical forces as drivers of cell fate, and in principal provides a framework for considering how development, adaptation, and evolution of palatogenesis can be influenced by unique feeding behaviors.

## Methods

### Animals

All experimental protocols followed ARRIVE (Animal Research: Reporting of *In Vivo* Experiments) guidelines and were approved by the Stanford Committee on Animal Research. Timed pregnancies were performed in all cases where embryonic mice were collected. C57BL/6 wild type embryos and mice were used for histological analyses and *Wnt1*^*Cre/+*^;*R26*^*LacZ/+*^embryos coupled with Xgal staining, were used to visualize cranial neural crest cells in the palate. In brief, Wnt1 is expressed in cranial neural crest cells as they emerge from the dorsal neural tube^49^; the second strain, ROSA26 conditional reporter (R26R), served as a substrate for the Cre-mediated recombination. Using this two-component genetic system, the migration and differentiation of cranial neural crest cells were followed using Xgal staining to detect the *LacZ* gene product. All animals were obtained from Jackson Laboratories.

### Histology, histochemistry, and immunohistochemistry

Tissues were fixed in 4% paraformaldehyde at 4 °C overnight and if needed, decalcified to completion in 19% EDTA at 23 °C, dehydrated in a graded ethanol series and embedded in paraffin or OCT. Coronal sections (8 μm) were cut and slide-mounted. In all cases the tissues analyzed spanned the region between the first and second molars.

Safranin O/Fast Green, Picrosirius Red, Aniline Blue, and whole-mount Alizarin red/Alcian blue stainings were used to identify bone and cartilage as described^50^. Histochemical stainings for Alkaline Phosphatase (ALP) and tartrate resistant acid phosphatase (TRAP) were performed as described^50^. Immunohistochemical localization of Ki67 (Thermo Scientific), Osteopontin (abcam), Collgen type X (abcam), and Sox9 (Santa Cruz Biotechnology) was performed as described^50^. For X-gal staining, cryosectioned slides were used; fixation, washing, and staining were performed as described^50^. TUNEL labeling was performed as described by the manufacturer (Roche). Imaging of stained tissue sections was performed with a Leica DM 5000B fluorescent microscope.

### Finite element modeling

Linearly elastic isotropic FE models were formulated in COMSOL Multiphysics 4.4 (COMSOL Inc.). The geometry used was based on histologic data from embryonic and postnatal midpalatal suture complexes (Table 1). The assigned mechanical properties of the palatine bone, soft connective tissue, and midpalatal suture were based on published reports (Table 2). The values assigned to the downward pressure caused by suckling^27^ and the upward pressure of tongue forces^26^ were estimated to be 1000Pa and 0.004N, respectively, using data obtained from human infants then scaled according to the weight of a mouse. In both the embryonic and postnatal models, the lateral edges of the palatine bones were constrained in their displacements in all directions.

All the experiments were carried out in accordance with the guidelines approved by the Stanford Committee on Animal Research.

## Additional Information

**How to cite this article**: Li, J. *et al.* Linking suckling biomechanics to the development of the palate. *Sci. Rep.* **6**, 20419; doi: 10.1038/srep20419 (2016).

## Supplementary Material

Supplementary Information

## Figures and Tables

**Table 1:** Tissue dimensions used in FE model construction.

Tissue	Thickness (mm)			
	Embryonic day 18	Postnatal day 1	Postnatal day 3	Postnatal day 7
Palatine bone	0.025	0.032	0.25	0.25
Dorsal connective tissue envelope	0.1125	0.071	0.05	0.05
Ventral connective tissue envelope	0.1125	0.071	0.05	0.05
Gap area	0.5	0.116	0.06	0.02
Cartilage cap	Not applicable	Not applicable	0.18	0.2

**Table 2:** Mechanical properties of tissues.

Tissue	Young’s elastic modulus (E)	Poisson’s ratio (ν)	References
Palatine bone	Prenatal = 100 KPa	Prenatal = 0.38	51
	Postnatal = 1000 MPa	Postnatal = 0.28	
Soft connective tissue	Prenatal = 50 KPa	Prenatal = 0.45	52
	Postnatal = 0.1 MPa	Postnatal = 0.45	
Cartilage	Prenatal = 50 KPa	Prenatal = 0.45	53
	Postnatal = 1 MPa	Postnatal = 0.45	
