# Supplementary material for: Linking suckling biomechanics to the development of the palate
Source: Sci Rep. 2016 Feb 4;6:20419. doi: 10.1038/srep20419 (PMC4740798; doi:10.1038/srep20419)
Supplement: Supplementary Information [file srep20419-s1.pdf]

Linking suckling biomechanics to the development of the palate

Jingtao Li[1,2], Chelsey A. Johnson[2,3], Andrew A. Smith[2], Daniel J. Hunter[2], Gurpreet Singh[2], John B. Brunski[2], and Jill A. Helms[2,§]

1 Department of Oral and Maxillofacial Surgery, West China Hospital of Stomatology, Sichuan University, Chengdu, China 610041

2 Division of Plastic and Reconstructive Surgery, Department of Surgery, Stanford School of Medicine, Stanford CA 94305

3 Department of Plastic Surgery, University of Pittsburgh Medical Center, Pittsburgh, PA 15261.

§ Corresponding author

Jill A. Helms, 257 campus drive, Stanford, CA, 94305

Email: jhelms@stanford.edu

Tel: 650 736 0919

FAX: 650 736 4374

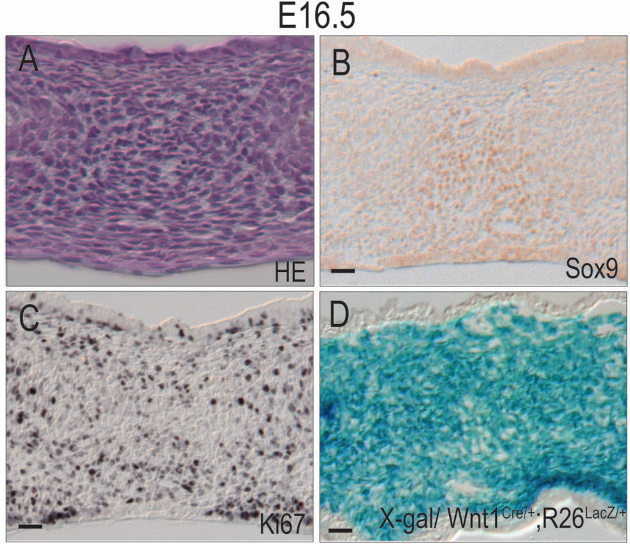

Supplementary figure 1

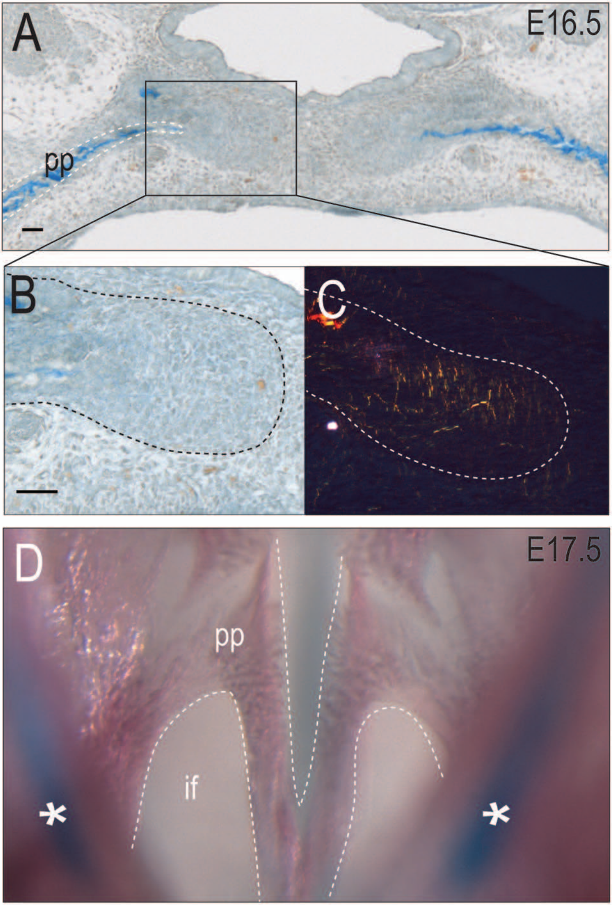

Supplementary figure 2

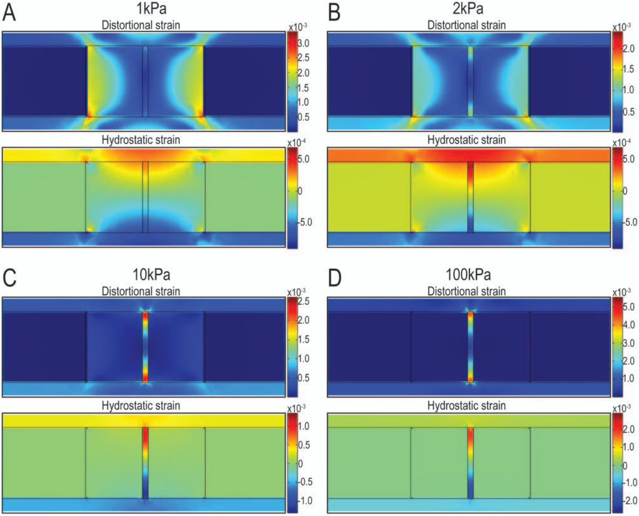

Supplementary figure 3

Figure Legends

Supplementary Figure 1. The homogenous tissue arragement of the palate during early embryogenesis.

(A) HE staining, and (B) Sox9, and (C) Ki67 immunostaining on representative tissue sections of the E16.5 palate. (D) X-gal staining of a representative tissue section from a E16.5 *Wnt1$^{Cre/+}$;R26$^{LacZ/+}$* embryo. Scale bar=50µm.

Supplementary Figure 2. Palatal bone forms through intramembranous osteogenesis.

(A) Aniline blue staining highlights the emerging palatal process of the maxillary bone (ppm) at E16.5 (outlined in white). (B) Higher magnification allows better visualization of the mesenchymal condensation that is forming around the leading edge of each ppm with Picrosirius red staining to the right (C) showing its composition being that of a fine collagenous extracellular matrix. (D) Alizarin red/Alcian blue staining of the mouse palate at E17.5. Abbreviation: pp=palatal process; if=incisive foremen. Asterisk indicates Meckel's cartilage. Scale bars=50 µm.

Supplementary Figure 3. Modeling the ossification of cartilage caps in the midpalatal sutue.

The distortional and hydrostatic strains were mapped as the cartilage was assigned progressively larger elastic moduli, from (A) 1kPa, (B) 2kPa, (C)10kPa, to (D) 100kPa, to simulate the gradual ossification of the cartilage cap.
